# Intrafamilial Genotyping of *Helicobacter pylori* from Faecal DNA

**DOI:** 10.1155/2011/491035

**Published:** 2011-07-31

**Authors:** M. McMillan, W. G. MacKay, C. L. Williams, A. J. Shepherd, C. Malcolm, L. T. Weaver

**Affiliations:** ^1^Child Health, School of Medicine, University of Glasgow, Glasgow G3 8SJ, UK; ^2^Department of Nursing and Midwifery, University of Stirling, Stirling FK9 4LA, UK

## Abstract

*Helicobacter pylori* infection, often acquired in early childhood, is a global cause of undernutrition, gastritis, peptic ulcer disease and gastric carcinoma. This study tested the feasibility of using *H. pylori* shed in the faeces as a source of DNA for non-invasive epidemiological studies. *H. pylori* DNA was chemically recovered and isolated using a specific biotinylated oligonucleotide probe with magnetic capture from 28 *H. pylori* positive faecal samples obtained from children attending hospital for the investigation of suspected *H. pylori* infection, together with close family members. Random amplification of polymorphic DNA (RAPD) was subsequently used to discriminate each isolate. 93% of stool samples selected were typeable. Parent, child and sibling samples were compared and similarities determined. Phylogenetic analysis showed that *H. pylori* DNA obtained from the faeces can be used to genotype individual strains, offering a means of studying intrafamilial transfer of this microorganism.

## 1. Introduction

The endemic gram-negative bacterium *Helicobacter pylori *colonises the gastric mucosa of up to half of the human population of the world [1]. Although in many cases it causes nonspecific dyspeptic symptoms or none at all, it is recognised that *H. pylori* is the principal cause of duodenal ulcer disease and can ultimately lead to the development of gastric cancer [2]. Knowledge of the full genome of *H. pylori *[3] makes it possible to isolate and genotype *H. pylori* DNA from colonized individuals for the purpose of studies of its distribution and transmission [4].

While many genotyping methods exist, they are generally performed on DNA samples obtained from the stomach by endoscopic gastric biopsy or aspiration. Such methods can be used to determine transmission routes and risk factors [4], but invasive methods of sampling are unsuitable for epidemiological studies involving young children [5]. Genotyping *H. pylori* DNA obtained from faecal samples offers an attractive non-invasive alternative method for such purposes.

While *H. pylori* is shed in the faeces, in the majority of cases, it is either difficult to culture or nonviable [6–8], and diagnosis is often made by serology based on the presence or absence of traces of the organism (antigen). This precludes analysis of the strains present [4]. We published a non-invasive method designed specifically to obtain *H. pylori* from human faeces [4] using a procedure for the organic extraction of DNA. It is necessary to show that purified *H. pylori* DNA extracted from faeces is intact genomic DNA which can be used for genotyping, offering a potential means of non-invasive determination of the transmission route of this microorganism.

Rapid amplification of polymorphic DNA (RAPD) has been used for epidemiological studies of local bacterial outbreaks [9]. Several have used RAPD fingerprinting of material obtained by gastric biopsy [10, 11] or gastric aspirates [12] to investigate the transmission of *H. pylori*, supporting the hypothesis of intrafamilial transmission. These studies indicated that there is a high level of sequence diversity within this microorganism; 64 independent *H. pylori* isolates were distinguishable in one study [10], and more recently a study of 32 cases suggested that family members have closely related *H. pylori* fingerprinting patterns [11]. Previously, RAPD analysis confirmed that children infected with *H. pylori* had strains identical to that of their mothers, consistent with vertical transmission from mother to child [12]. RAPD is highly discriminatory and, therefore, useful in the investigation of short term or local outbreaks of disease [10–12]. This paper describes a method for extraction and isolation of DNA from human faeces followed by molecular genotyping. RAPD was evaluated on samples obtained from infected children and their immediate family members.

## 2. Subjects, Materials, and Methods

### 2.1. Subjects

**Figure 1:**
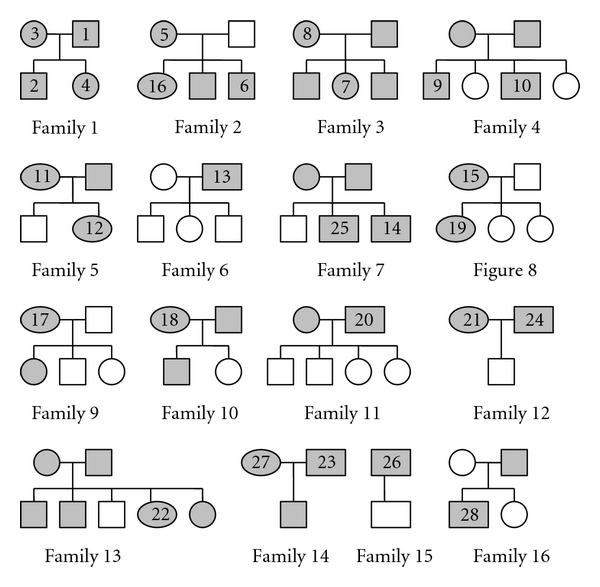
Family trees showing *H. pylori* positive study participants who provided faecal samples. Circles represent females, and squares represent males. Older generations are represented above younger ones. Unshaded symbols represent family members who tested antigen negative while shaded symbols represent those who tested antigen positive. Numbered symbols denote subjects from whom sufficient stool remained after HpSA testing to undergo *H. pylori* genotyping. Adapted from family tree data (13).

Children (aged up to 16 years) attending the Royal Hospital for Sick children (Glasgow, UK) for urea breath test (UBT) of suspected *H. pylori* infection were studied. Those with positive UBT results, together with close family members, were asked to provide faecal samples. These were obtained after informed parental consent and underwent HpSA analysis to determine their *H. pylori* status [13]. Twenty-eight positive faecal samples from children and family members were selected for this methodological study (Figure 1). In some cases insufficient stool was available after HpSA analysis to permit DNA extraction and genotyping.

### 2.2. Urea Breath Tests

Duplicate fasting baseline breath samples were collected by exhaling through a straw into an exetainer (Labco, High Wycome, UK) before and 30 min and at 40 min after oral ingestion of 20 mls of 15% polycose (Abbott Laboratories, Dublin, Ireland), containing 100 mg ^13^C urea (99 atom% excess; CK Gas, Berks, UK). The abundance of breath ^13^C was measured by continuous-flow isotope ratio mass spectroscopy (20/20 PDZ Europa, Crewe, UK). Enrichment was calculated as excess abundance of the 30 min after ^13^C urea dose over the baseline reading. A reading of 40.0 ppm (delta over baseline, 3.5%  ^13^C) was determined diagnostic of *H. pylori *colonisation [14].

### 2.3. HpSA Analysis

Faecal samples were frozen at −80°C until analysis. An enzyme immunoassay (Premier Platinum HpSA, Meridian Diagnostics Inc., Cincinnati, USA) was used to determine the presence or absence of *H. pylori* antigen. This serological test relies on the binding of *H. pylori* to anti-*H. pylori* antibodies on microwells in the presence of peroxidase-conjugated polyclonal antibodies [13]. Incubation for 1 h at 24°C was followed by washing to remove unbound material then incubation with substrate for 10 min. The reaction was terminated by the addition of sulphuric acid stop solution. Absorbance was measured spectrophotometrically at 450 nm (Multiscan Acent; Thermo Life Sciences, Basingstoke, UK) [13].

### 2.4. Validation of Integrity of *H. pylori* DNA after Passage through Gut

Faecal samples previously collected from two adults (one positive and one negative) were treated as for DNA extraction by centrifugation and then divided into five 1 mL fractions (A to E) to determine quantities of culturable bacteria and the presence of *H. pylori* antigen. These consisted of two samples from the creamy layer (D and E) and three samples from the supernatant above (A to C). The creamy layer had previously been shown to contain a concentrated mass of bacterial cells [7]. A modified Miles and Misra plate count was used to perform cultural analysis [4]. The faecal fractions were serially diluted in PBS, and 20 uL volumes were plated in triplicate onto predried Wilkin-Chalgren anaerobic agar plates (Oxoid Ltd., UK) and incubated at 37°C for 24 hr in an anaerobic gas jar (Beckton Dickinson UK Ltd., UK). The detection of *H. pylori* antigens was performed using the HpSA enzyme immunoassay.

### 2.5. Faecal DNA Extraction and Polymerase Chain Reaction

Six grams of defrosted faecal sample were homogenised for 2 min at room temperature with phosphate-buffered-saline (0.01 M phosphate buffer, 0.0027 M potassium chloride, 0.317 M sodium chloride [pH 7.4] (Sigma-Aldrich, Poole, UK) using a Stomacher 400 (Seward Medical, London, UK). The resultant 20% (wt/vol) faecal slurry was centrifuged at 20,000 ×g for 30 min (Heraeus Biofuge Stratos centrifuge; Kendro Laboratory Products, Sollentum, Germany) to concentrate the faecal flora [4]. After the removal of the supernatant with a plastic Pasteur pipette, the creamy phase boundary was removed to a separate 2 mL eppendorf (Fisher Scientific UK, Leicester, UK) where an equal volume of Prepman Ultra was added (Applied Biosystems, Cheshire, UK). This reagent was manufactured for the specific isolation of PCR quality DNA from foodstuffs and used according to the manufacturer's instructions.

Samples were boiled at 100°C for 10 min in a water bath to denature proteins and break up the bacterial cell walls before cooling for 2 min and centrifugation at 20,000 ×g for 10 min (Heraeus Biofuge Stratos) to pellet the particulates. Organic extraction using Phenol: Chloroform: Isoamylalcohol (PCI) 25 : 24 : 1 (Sigma-Aldrich, Poole, UK) was employed to extract total nucleic acids, which involved the mixing of equal volumes of PCI and faecal supernatant by inversion before centrifugation at 20,000 ×g for 5 min (EBA 12 Centrifuge, Hettich Zentrifugen, Tuttlingen, Germany). The nucleic acid containing upper aqueous phase was transferred to a fresh 2 mL eppendorf tube, and the process repeated twice. Traces of PCI were finally removed in a similar fashion to the above procedure but using chloroform (Sigma Aldrich, Poole, UK). Total nucleic acids were recovered by ethanol precipitation using 0.1 volumes of ice cold 3 M sodium acetate and two volumes of 100% ethanol (Sigma-Aldrich, Poole, UK). Following mixing and incubation on ice for 30 min, samples were centrifuged at 16,000 ×g for 15 min (EBA 12 centrifuge, DJB Labcare Ltd., Newport Pagnell, UK). After the removal of supernatant, the samples were allowed to air dry for 30 min before resuspension in 266 *μ*Ls of TE buffer (Sigma-Aldrich, Poole, UK).

RNA was degraded by incubating with 40 U of RNAse One (Promega, Southampton, UK) at 37°C for 1 h before the enzyme was removed by ethanol precipitation as previously described. Contaminating DNA was removed by gene capture using a biotinylated oligonucleotide probe to a specific region of the 16SrRNA gene which in *H. pylori, *as in common with all unique bacterial species, displays little allelic variation. This is so despite specific *H. pylori* genes being highly polymorphic with a mean genetic diversity of 0.83 exceeding that for all other bacterial species [2]. This involved the binding of a specific capture probe (5′ biotinylated probe: capC [5-GGG GAG TAC GGT CGC AAG ATT AAA ACT CAA AGG AAT A-3]) (Sigma-Aldrich, Poole UK) [15] to the *H. pylori *DNA followed by overnight hybridisation. Streptavidin-coated magnetic beads were then incubated with the hybridised complex to allow biotin-streptavidin interaction. *H. pylori* DNA was separated by electromagnetic separation from the faecal flora and inhibitory compounds that might have interfered with subsequent PCR [4]. DNA samples were stored at −20°C until analysis.

### 2.6. RAPD Analysis

A single arbitrary primed PCR primer (1254) with sequence [5-CCGCAGCCAA-3] [10] (Sigma-Aldrich, Poole, UK) was used to amplify segments of isolate DNA. This allowed the visual comparison and photography of agarose gel electrophoresis banding patterns between isolates using Quantity One software (Bio-Rad Laboratories Ltd., Hemel Hempstead, UK). All experiments used a positive control (purified *H. pylori* DNA strain NCTC 26695) and a negative control (faecal sample negative for *H. pylori *by HpSA), processed identically to study samples. All samples, including controls, were run in duplicate.

Amplification of gene fragments was performed with a PTC-200 Peltier thermal cycler (MJ Research, Waltham, Massachusetts, USA). PCR amplification was performed using a total of 50 *μ*L reaction volume, containing 1 *μ*L of template, 1 × buffer, 3 mM MgCl_2_, dNTP 250 *μ*M each of dATP, dTTP, dGTP and dCTP, 0.4 *μ*M primer and 1.0 U of taq DNA polymerase per reaction volume (Abgene Ltd., Epsom, UK). This was modified from the method previously described [10]. Thermocycling was performed under the following conditions: 95°C for 15 min, 94°C for 5 min, 36°C for 5 min 72°C for 5 min repeated four times, followed by 94°C for 1 min, 36°C for 1 min, and 72°C for 2 min repeated 30 times. A final 10 min at 72°C was used to finish as described previously [10]. BioNumerics software (Applied Maths NV, Sint-Martens-Latern, Belgium) was used to assess the similarity between lanes using the Dice coefficient [16] for analysis of RAPD genetic fingerprints of *H. pylori* strains produced by gel electrophoresis. RAPD patterns were normalised using a 0.1–1 kpb molecular size standard (Abgene Ltd., Epsom, UK), and UPGAMA cluster analysis was performed.

## 3. Results and Discussion

### 3.1. Analysis of Culturable Bacteria and *H. pylori* Antigens

**Figure 2:**
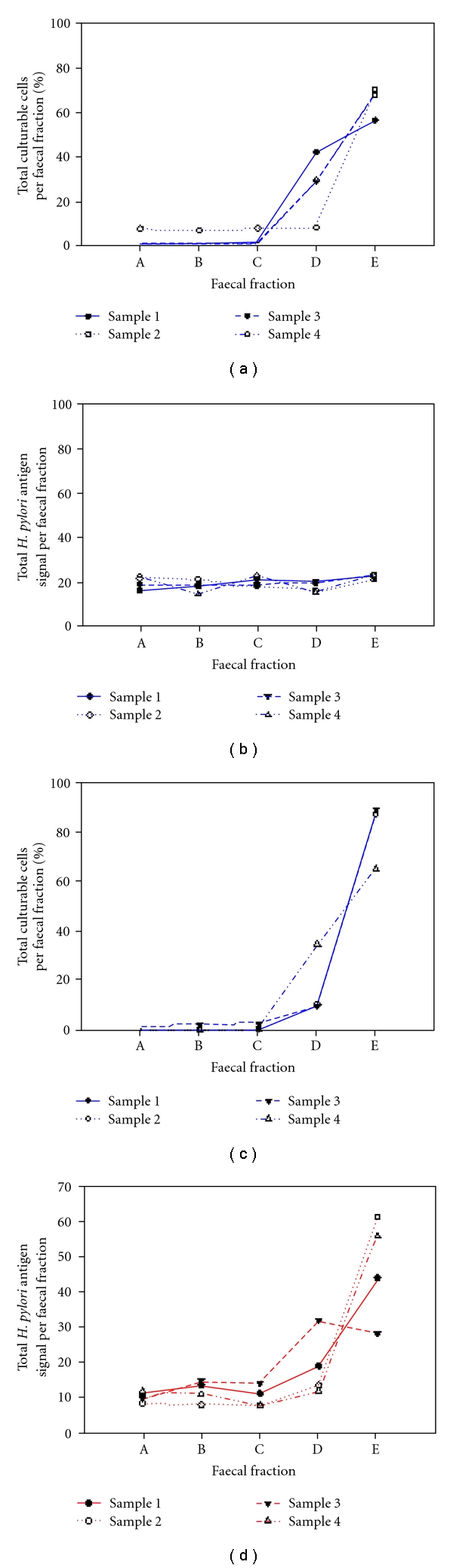
The percentage of total culturable bacterial cells (a, c) and *H. pylori* antigen signals (b, d) per faecal fraction. The data shown at (a, b) are for the *H. pylori*-negative subject and at (c, d) for the *H. pylori-*positive subject.

In the *H. pylori *negative adult subject, culturable bacterial load increased with fraction density from a mean of 8.25 × 10^8^ CFU/mL faecal fraction (SE 2.3 × 10^7^) in fraction A (9.6% total CFU/mL) to a mean of 6.2 × 10^9^ CFU/mL faecal fraction (SE 3.6 × 10^9^) in fraction E (65.1% total CFU/mL). In the *H. pylori* positive subject, the culturable bacterial load increased with fraction density from a mean of 4.7 × 10^6^ CFU/mL faecal fraction (SE 2.5 × 10^6^) in fraction A (0.87% total CFU/mL) to a mean of 6.8 × 10^8^ CFU/mL faecal fraction (SE 2.6 × 10^8^) in fraction E (82.5% total CFU/mL) [4]. The numbers of culturable bacteria were in both cases proportional to the density of the fraction. For both individuals, the majority of all culturable bacteria were in fraction E (Figure 2). 

Antigens were detected only in the creamy layer of the faeces of the *H. pylori* colonized individual (mean OD_450_ 1.3, SE 0.4 (negative < 0.10, equivocal ≥ 0.1 < 0.12, positive ≥ 0.12)). In the *H. pylori*-colonized individual, the antigen load increased with fraction density from a mean of 10.5% total antigen in fraction A to a mean of 47.5% in fraction E (SE 7.33) (Figure 2) suggesting that *H. pylori* passes through the gut intact, since the highest *H. pylori* antigen signal was found in the faecal fraction that contained the majority of the culturable bacteria [4]. 

### 3.2. Limit of Detection of RAPD PCR

**Figure 3:**
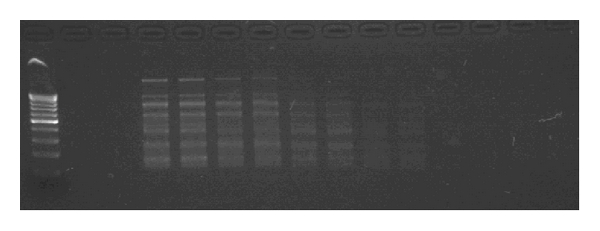
Limit of detection of *H. pylori* RAPD. This photograph displays a series of sequential dilutions of a 16.16 ugs/mL DNA concentration. Detection of bands is good at dilutions equivalent of 2.17 × 10e^6^ *H. pylori* bacterium.

A series of dilutions was made of a 16.16 ugs/mL stock DNA solution. This was then used for PCR amplification in duplicate, and a gel was run of the PCR products. The limit of detection was determined to be the dilution of the stock solution amounting to 8.08 ugs/mL, equivalent to 2.17 × 10e^6^ bacteria (Figure 3).

### 3.3. Results from Subject Samples

**Figure 4:**
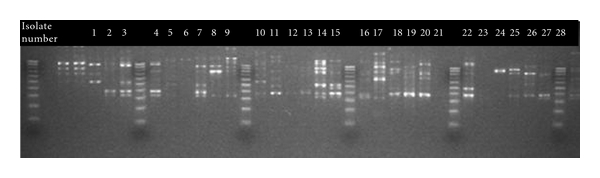
RAPD typing of 28 *H. pylori *isolates. Analysis of banding patterns of 2% agarose gel electrophoresis using Quantity-One imagining software v 4.5.0 (Bio-Rad Laboratories Ltd.). Lane 1, 8, 15, 22, 29, and 36 (DNA 100 bp ladder 100–1000 bp), lane 2 (negative control), lane 3 and 4 (positive culture controls), Lane 5–7, 9–14, 16–21, 23–28, 30–35, and 37 (isolates 1–28).

**Figure 5:**
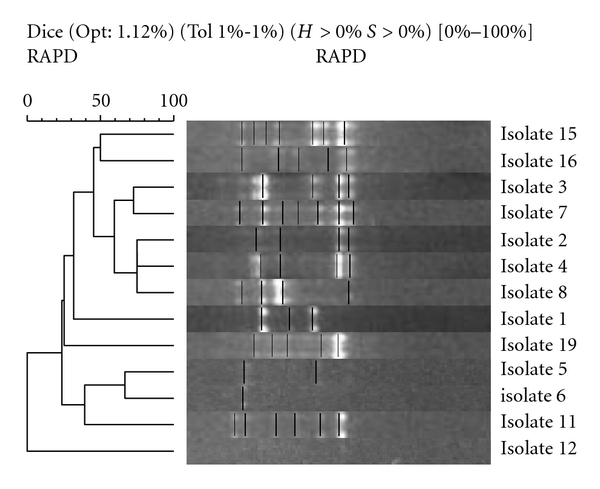
Parent and child isolates: neighbour-joining phylogenetic analysis of RAPD amplification of isolates using Gelcompar II software (Applied Maths NV, Sint-Martens-Latern, Belgium). This is a schematic representation of sample similarity: the gel bands of each isolate (middle) are represented by vertical lines and have been aligned to correspond with their position in the tree (left). This tree represents the minimal total branch length at each stage of clustering. The approximate distance between any pair of samples is calculated from the similarity matrix (not shown).

Twenty-six of the 28 *H. pylori* study isolates that underwent RAPD typing produced reproducible banding patterns, giving a typeability of 93%. Of these isolates, the (1254) primer produced bands that were highly discriminatory in all cases where there were electrophoresis bands present. No two samples were identical, and each varied between a high of seven and a low of one band.  Figure 4 shows the banding patterns with 2% agarose gel electrophoresis using Quantity-One imagining software v 4.5.0 (Bio-Rad Laboratories Ltd.). RAPD electrophoresis banding patterns from families which had parent and child isolates were compared in Figure 5. 

**Figure 6:**
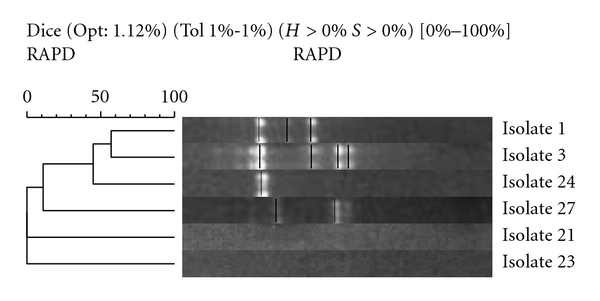
Parental samples: neighbour-joining phylogenetic analysis of RAPD amplification of isolates using Gelcompar II software (Applied Maths NV, Sint-Martens-Latern, Belgium).

All electrophoresis banding patterns gave a low degree of homology. In Family 1, the mother's sample (isolate 3) was slightly more closely related to the children's samples (isolates 2 and 4) than the father's sample (isolate 1), 60% compared with 40%. In Family 2, samples (isolates 5 and 6) bore a similarity of 65%, while in family three, isolates derived from mother and daughter (isolates 7 and 8) showed a degree of homology with a similarity of 60%. All other parent and sibling samples showed less than 50% similarity. Isolates from parental stool samples are compared in Figure 6. 

**Figure 7:**
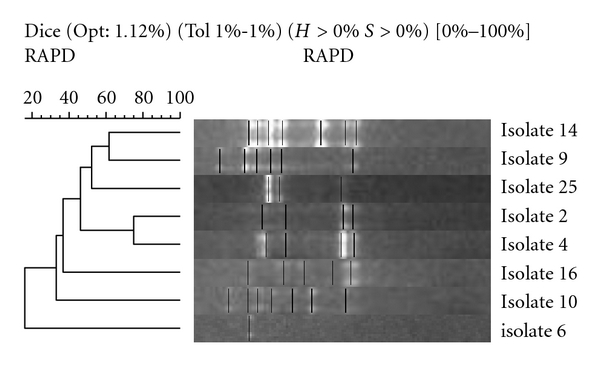
Sibling samples: neighbour-joining phylogenetic analysis of RAPD amplification of isolates using Gelcompar II software (Applied Maths NV, Sint-Martens-Latern, Belgium).

**Figure 8:**
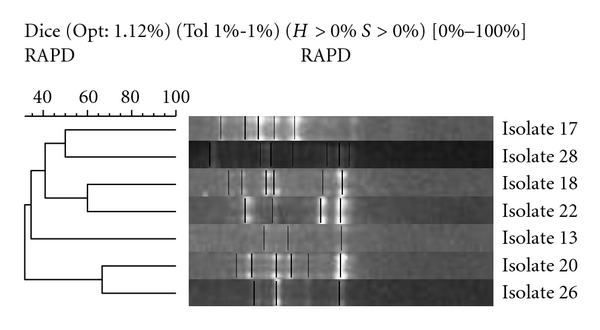
Single samples: neighbour-joining phylogenetic analysis of RAPD amplification of isolates using Gelcompar II software (Applied Maths NV, Sint-Martens-Latern, Belgium).

Isolates [1 and 3] from Family 1 had a similarity of 55%, while samples (isolates 21 and 23) failed to produce banding patterns and could not be compared with samples (isolates 24 and 27), respectively. Similarities between siblings were found to be mixed (Figure 7). Isolates 2 and 4 from family one have a similarity of 78%, while all other sibling samples (isolates 6 and 16, 9 and 10, and 14 and 25) showed similarities below 50%. All single-member family isolates had low similarity when compared with each other (Figure 8). The closest similarity between isolates from single unrelated members of the cohort was 62% between isolates 20 and 26, 60% between isolates 18 and 22, and 50% between isolates 17 and 28. All others were below 50% similarity.

## 4. Discussion

Previous studies have shown that *H. pylori* DNA obtained from faecal samples can be used to successfully genotype *H. pylori* DNA [4, 15]. This paper describes a method for genotyping *H. pylori* by coupling the extraction of intact genomic DNA shed into the faeces of infected individuals with the highly discriminatory method of RAPD. We have shown that *H. pylori* passes through the gut intact by demonstrating that HpSA antigen detection is greatest in the centrifuged fraction where the greatest number of culturable bacteria are located. Genotyping of DNA recovered will, therefore, be a valid representation of genotyping the whole bacterial genome, and not fragments, which would otherwise have distorted the typing results.

RAPD has proved to be an invaluable aid in the epidemiological study of pathogenic bacteria in outbreaks [16, 17], allowing the molecular characterisation of *H. pylori* [18]. Previous studies involving closely related *H. pylori* strains obtained from gastric biopsies have shown intrafamilial dispersion using RAPD fingerprinting [11]. RAPD genotyping of *H. pylori* DNA derived from faecal samples discriminated between all isolates that were typeable giving unique sets of banding patterns. 

In our study, similarities were low between all isolates; the two NCTC 26695 positive control lanes were 90.9% similar which was the maximum similarity obtained. Most of our isolates gave a similarity of lower than 65% regardless of whether the donors were related to each other or not. Interestingly however, in Family 1, the two sibling samples gave a similarity of 78% which points towards some degree of homology between these isolates.

It was disappointing that not all HpSA-positive faecal samples were of sufficient volume to permit DNA extraction. Nevertheless, only two out of the 28 isolates tested failed to give any type of genetic fingerprint. Some isolates produced many bands while others only a few or even only one. One consequence of this variation in the amount of *H. pylori* isolated from each sample was that it may have lead to differing intensity of RAPD band sizes even in clonal isolates. Fainter bands not detected by Bionumerics software could lead to the generation of differences in similarities between lanes. This could be eliminated in future studies by using greater volumes of faecal material and standardizing the *H. pylori* DNA extract before commencing the RAPD PCR. 

Previous studies using RAPD analysis suggest that there is a rapid accumulation of genetic variation in *H. pylori *[17]. In shorter term epidemiological studies, extensive genetic variation within the pathogen of interest indicates that it will be unlikely that any two patients will be colonised independently with strains that give the same electrophoresis patterns by chance. Individuals infected with strains giving the same banding patterns are, therefore, likely to share a common source of infection [17]. The source of *H. pylori* DNA was restricted to faeces owing to ethical and practical constraints. It is neither clinically permissible nor practically possible to obtain gastric material from large numbers of subjects, including children, within epidemiological studies of families.

The identification of slight differences between DNA fragments within clonal groups is necessary to trace patterns of transmission in epidemiological studies. Differentiation of unrelated isolates (discriminatory power), as well as unambiguously providing a result for each isolate analysed (typeability), are important performance criteria in any typing system [19]. In longer-term epidemiological studies, too rapid accumulation of variation is a distinct disadvantage as it could give rise to no recognisable electrophoresis patterns as all clonality is destroyed [17]. RAPD typing provides a method of easily producing typeable data based on familial groupings over a short time span. Variation is likely to occur between samples as *H. pylori* undergoes considerable genetic mutation and recombination due to the pandemic nature of this species [20].

## 5. Conclusions

This is the first description of a method that combines RAPD fingerprinting and faecal *H. pylori* DNA extraction using gene capture. RAPD results are promising in that isolates from 93% of subjects gave genetic fingerprints. The use of greater volumes of faecal material for extraction purposes together with standardisation of extracted *H. pylori* DNA before RAPD PCR might improve sensitivity. This would help to confirm similarities between clonal isolates, and, therefore, we would recommend this practice in further studies. However, we have provided evidence that *H. pylori* DNA obtained from faecal samples contains intact genomic DNA, and the development of the methods described offers a promising means of studying the intrafamilial transmission of this ubiquitous microorganism.

##  Conflict of Interests

The authors declare that there is no conflict of interests.
